# Smoking Attributable Risk in Multiple Sclerosis

**DOI:** 10.3389/fimmu.2022.840158

**Published:** 2022-03-03

**Authors:** Ali Manouchehrinia, Jesse Huang, Jan Hillert, Lars Alfredsson, Tomas Olsson, Ingrid Kockum, Cris S. Constantinescu

**Affiliations:** ^1^Centre for Molecular Medicine, Karolinska Institutet, Stockholm, Sweden; ^2^Department of Neurology, Cooper Neurological Institute, Camden, NJ, United States; ^3^Section of Clinical Neurology, Academic Division of Mental Health and Clinical Neuroscience, University of Nottingham, Nottingham, United Kingdom

**Keywords:** multiple sclerosis, attributable fraction, risk factor, tobacco, smoking

## Abstract

Tobacco smoke is an important modifiable environmental risk factor for multiple sclerosis (MS) risk. The population attributable fraction (AF) of MS due to smoking can be used to assess the contribution of smoking to the risk of MS development. We conducted a matched case-control study, including individuals with MS and population-based controls. Overall, sex- and genetic risk score-stratified AF due to smoking were calculated by fitting logistic regression models. We included 9,419 individuals with MS and 9,419 population-based matched controls. At the time of MS onset 44.1% of persons with MS and 35.9% of controls ever regularly smoked of which 38.1% and 29.2% were still smoking. The overall AF was 13.1% (95%CI: 10.7 to 15.4). The AF was 10.6% (95%CI: 7.4 to 13.7) in females and 19.1% (95%CI: 13.1 to 25.1) in males. The AF was 0.6% (95%CI: 0.0 to 2) in ex-smokers. In those having human leucocyte antigen (HLA) and non-HLA risk scores above the median levels of controls, the AF was 11.4% (95%CI: 6.8 to 15.9) and 12% (95%CI: 7.7 to 16.3), respectively. The AF was 17.6% (95%CI: 10.2 to 24.9) and 18.6% (95%CI: 5.5 to 31.6) in those with HLA and non-HLA risk scores below the median levels in controls, respectively. We noticed a decline in AF in recent birth cohorts. This study indicates that at least 13% of cases of MS could be prevented through the avoidance of tobacco smoking. Considering the prevalence of MS, this represents a very large group of people in absolute number.

## Introduction

Multiple sclerosis (MS) is the result of a complex interplay between genetic and environmental risk factors. Prevention of MS is becoming a reasonable aspiration, but it depends on the extent risk factors can be modified or mitigated. Cigarette smoking remains the most important single cause of preventable mortality and morbidity in the world. Exposure to cigarette smoke is also an important modifiable environmental risk factor for MS development and its clinical course (1–4), with epidemiological studies reporting a 50% higher risk of MS development in ever-smokers compared to never-smokers. (5)

The population attributable fraction (AF) of disease due to exposure, or the proportion of the disease in a population attributable to smoking, can be used to assess the contribution of smoking to the risk of disease development. Estimating the AF could be a strong incentive to prevent disease by measuring the population burden associated with a given exposure. For example, the AF for smoking in people with lung cancer is 85% (6), indicating that a substantial number of lung cancers would not have occurred and relatively less lives would be lost if people did not smoke.

For smoking-associated diseases such as MS, the smoking AF increases with a higher prevalence of smoking in the population. Estimating the AF of MS due to smoking would determine the proportion of MS that could be avoided if people did not smoke. In this study, we estimated the AF due to smoking of MS in the Swedish population.

## Method

### Data Source

Our study population included individuals with MS and population-based controls participating in two large Swedish cohorts, the Epidemiological Investigation of Multiple Sclerosis (EIMS) and the Genes and Environment in Multiple Sclerosis (GEMS). (7) In the EIMS study, individuals with newly diagnosed MS (fulfilling the McDonald criteria), aged between 16 and 70 years, were identified at neurology clinics throughout Sweden and invited to participate in the study by completing a questionnaire and donating a blood sample. (8) The GEMS study includes prevalent cases of MS fulfilling the McDonald criteria who were identified from the Swedish National MS registry. (9) There were no overlap of cases between EIMS and GEMS. Controls were randomly selected from the national population register matched for age (equivalent of age at the diagnosis in cases), gender, and residential area at the time of the disease diagnosis. In both studies, information on exposure to tobacco smoking was obtained by asking about current and previous smoking habits. We defined “smokers” as those who have ever smoked cigarettes regularly before MS onset or the equivalent age in controls.

### Adjustment for Genetic Risk Score

For a subset with available genetic data, the weighted human leucocyte antigen (HLA) specific and non-HLA genetic risk scores were calculated. Individuals were genotyped using an Illumina custom array (>90,000 SNPs), which focuses on MS genetic risk loci, particularly the HLA region on chromosome 6. (10) Standard marker and individual quality controls were performed using PLINK, and population outliers were identified using SmartPCA. After quality control and exclusion of outliers, classical HLA allele variants were imputed using HLAIMP*03. (10) Each individual’s genetic susceptibility to MS was determined using a polygenic risk score (GRS) defined by the sum of all risk alleles carried (0, 1, and 2). Total risk and separated scores for HLA allele variants and non-HLA SNPs were calculated using established risk factors from previous interaction and genome-wide association studies. (10, 11) Scores were also weighted by their effect size controlled for population stratification and possible confounding genetic factors.

### Statistical Analysis

For the calculation of AF, we used the method suggested by Dahlqwist et al. (12) In short, the method calculates confounder adjusted AF estimates for case-control study design. For each case of MS we identified one exact calendar year of birth and sex matched control while adjusting all the models for calendar year of birth in five groups. This was mainly done to account for changes in the prevalence of smoking in the Swedish general population over the years. Overall and sex stratified AF were calculated in the first instant. We also calculated AF stratified by HLA and non-HLA genetic burden. The genetic risk scores were dichotomized by the median score of the population-based controls. In a subset of individuals with information on HLA DRB1*15:01, we calculated smoking AF due to interaction (carriage of HLA DRB1*15:01 and being a smoker).

## Results

We included 9,419 individuals with MS and 9,419 population-based exact calendar year of birth and sex matched controls. 28% of the population were male with mean (standard deviation, SD) calendar year of birth of 1960 (± 14). 44.1% of persons with MS and 35.9% of controls had ever-smoked prior to onset or index age respectively. At the time of MS onset (and equivalent time in controls) 38.1% of cases and 29.2% of controls were still smoking (current smokers). The mean number of pack-years cigarette smoked was significantly higher in cases compared to controls [4.2 (± 7.2) vs. 3.2 (± 6.7), *P* < 0.001]. Cases smoked on average 5.7 (± 7.5) cigarettes per day for the duration of 6.2 (± 9.1) years. The average number of cigarettes smoked was 4.4 (± 6.9) for the duration of 4.9 (± 8.6) years in controls. The HLA and non-HLA genetic risk scores were available in 5,916 controls and 6,885 persons with MS (**Figure 2**). The risk of MS in ever-smokers was increased by 41% (95% confidence intervals (CI): 1.33 to 1.50) compared to never smokers.

**Figure 1:**
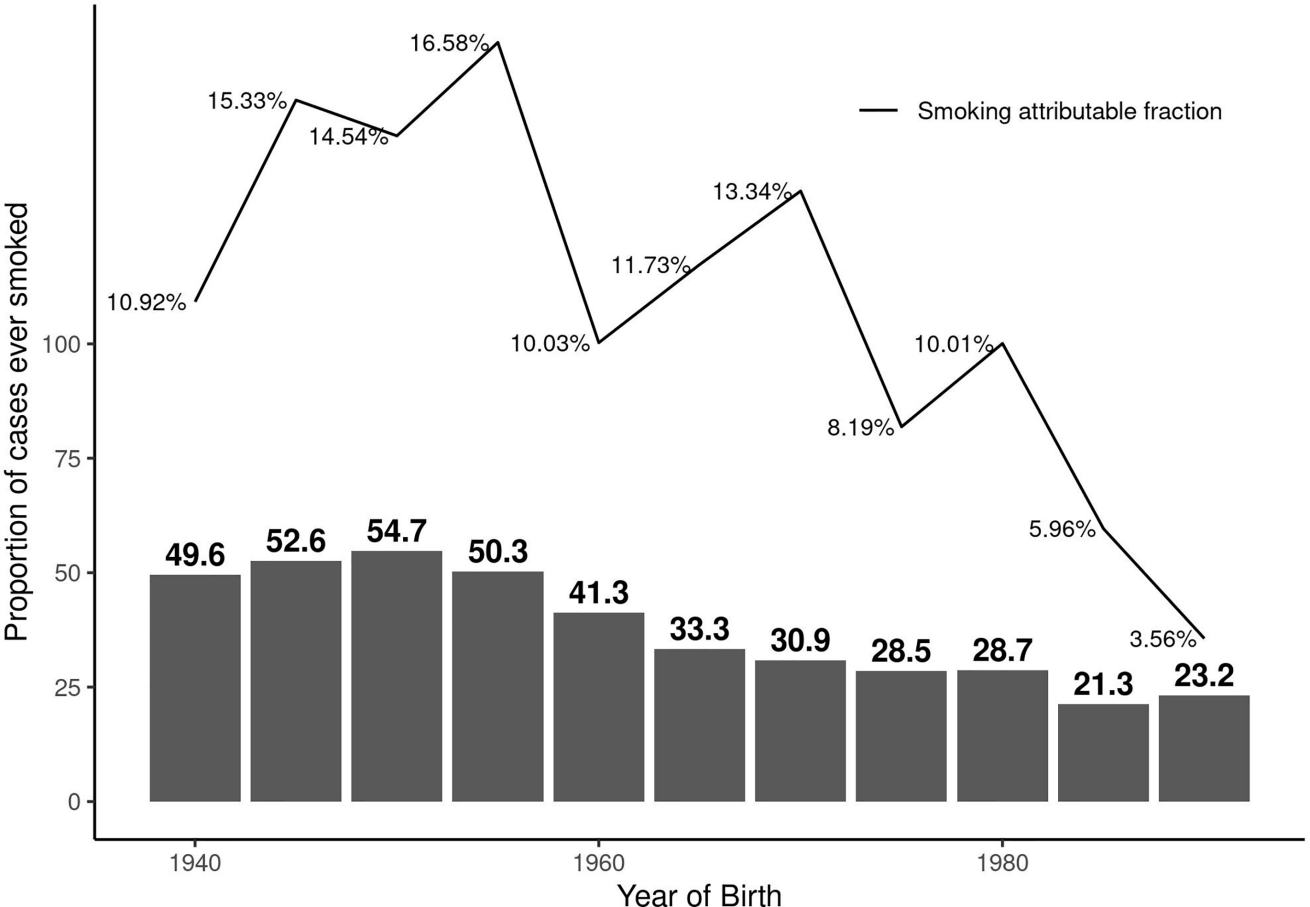
Proportion of persons with MS who have ever regularly smoked and the corresponding smoking attributable fraction (AF) over calendar year of birth by five-year birth-cohort.

The overall AF was 13.1% (95%CI: 10.7 to 15.4). The AF was 10.6% (95%CI: 7.4 to 13.7) in females and 19.1% (95%CI: 13.1 to 25.1) in males. The AF was less than 1% (AF: 0.6%, 95%CI: 0 to 2) in ex-smokers indicating beneficial effects of smoking cessation. **Figure 1** illustrates the AF over five-year birth-cohort strata along with percentage of smokers amongst cases in each birth-cohort. When investigating the impact of smoking intensity, we observed that the AF was 9.3% (95%CI: 7.1 to 11.5) in those smokers with pack-years smoked above the median and 7.1% (95%CI: 4.9 to 9.3) in those who smoked below the median smoking intensity.

**Figure 2:**
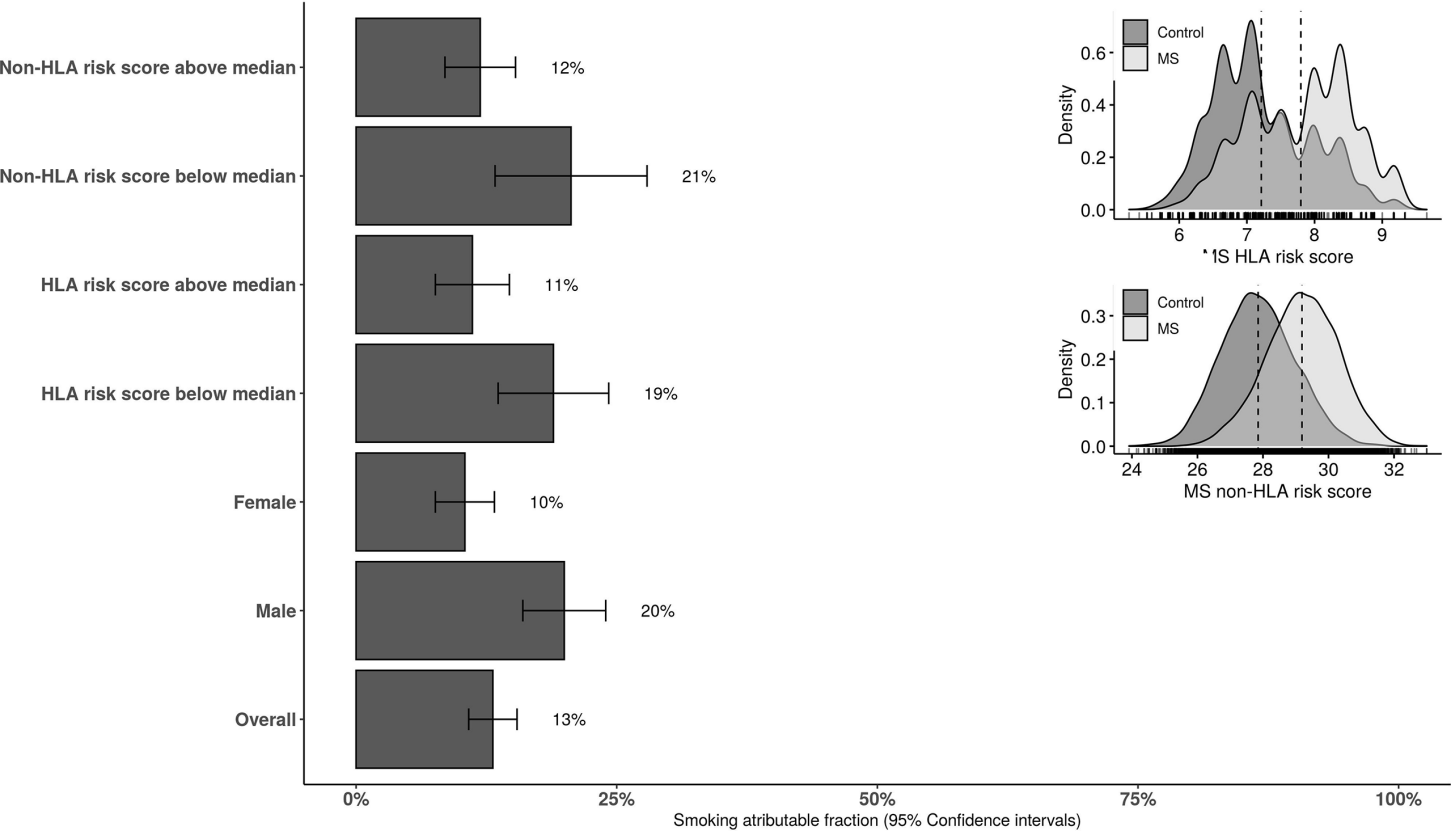
The overall and stratified smoking attributable fraction (95% confidence intervals) for risk of MS. HLA and non-HLA risk scores are categorized to below and above the median score in the population-based controls. HLA, human leucocyte antigen.

In those having HLA and non-HLA risk scores above the median levels of controls, the AF was 11.4% (95%CI: 6.8 to 15.9) and 12% (95%CI: 7.7 to 16.3), respectively. The AF was 17.6% (95%CI: 10.2 to 24.9) and 18.6% (95%CI: 5.5 to 31.6) in those with HLA and non-HLA risk scores below the median levels in controls, respectively. The stratified AF estimates are summarized in **Figure 2**. Results of interaction analysis between HLA DRB1*15:01 and smoking revealed that approximately half of the AF due to smoking is independent of HLA DRB1*15:01 status (6.8%, 95%CI: 5.5 to 7.9) while the AF due to HLA independent of smoking was 23% (95%CI: 22.1 to 23.8). The risk (OR) associated with both DRB1*15:01 carriage and smoking was 4.9 (95%CI: 4.4 to 5.4) compared to 3.4 (95%CI: 3.1 to 3.8) and 1.5 (95%CI: 1.4 to1.7) for only DRB1*15:01 carriage and smoking, respectively. AF in MS cases due to having both exposures was 20% (95%CI: 19.4 to 20.5).

## Conclusion

There is strong evidence for a link between tobacco smoking and the risk of MS, and data support the concept that this link is most likely causal. (13) Based on this concept, this study indicates that at least 13% of cases of MS could be prevented through the avoidance of tobacco smoking. Considering the prevalence of the disease, this represents a very large group of people in absolute numbers who would never develop MS. We believe that, from a global perspective, these numbers may represent an underestimate. The study was based on a well-characterized sample of Swedish population. In Sweden, the prevalence of MS is high, and the prevalence of smoking is low. (14) As the AF increases with the prevalence of smoking, we believe that the AF in a country with a high prevalence of smoking could be considerably higher.

Our previous estimates of smoking AF in MS was 20% (active or passive) which was further increased to 41% in subjects who had carriage of HLA-DRB1*15 and absence of HLA-A*02. (15) This study includes a much greater number of cases and controls compared to our previous study. We also used polygenic risk scores and specific risk alleles (HLA DRB1*15:01) which provide additional information with regard to the interaction between smoking and MS genetics. We previously estimated that about 5% of the MS cases could be attributed to passive smoking. (15) Passive smoking (for example, exposure to parental smoking in childhood) has not been included in the present analysis. However, we could confirm that smoking cessation and to lesser extent reducing smoking intensity could be potentially beneficial in reducing the impact of smoking in MS. We also observed a decline in smoking AF in a more contemporary cohorts of MS as the smoking prevalence declines in the general population. Given that the majority of environmental risk factors for MS tend to exert their most powerful effect in childhood and adolescent years and that parental smoking is a risk factor for MS, it is possible that a further fraction of MS could be prevented in the offspring of smokers. Although an AF of MS due to passive smoking cannot be calculated in the current study, this consideration reiterates the possibility that the 13% of MS attributable to smoking is underestimated.

Smoking remains a major modifiable risk factor for many morbidities, including MS. The association of smoking with a worsened outcome in multiple conditions, including MS, (16) has incentivized smoking cessation. In this regard, several smoking cessation campaigns have been effective in reducing smoking in the population, encouraging cessation. (17) If a smoking avoidance campaign has similar rates of success, an important proportion may avoid MS.

Our previous studies addressed the interaction between MS risk factors such as smoking, HLA DRB1*15:01, obesity, sun exposure, and EBV seropositivity. The interaction of HLA DRB1*15:01 with smoking increased the combined AF for MS. Here, we used a combined genetic risk scores which does not take only HLA DRB1*15:01 into account, and find a slightly less multiplicative increase when population was stratified by their MS genetic susceptibility. We consider a smoking AF of 13% an accurate overall estimate, but are aware that the interaction of smoking with other risk factors is complex. For example, smoking has complex, stimulatory effects on the immune system (4) and can enhance inflammatory contributors such as EBV and counteract protective factors such as sun exposure. Smoking has also shown to be associated with the worsening of disability in MS (1) and smoking cessation to be beneficial (2, 18). To date, no study has investigated the smoking AF with regard to disability accumulation in MS. Such study could be highly valuable in informing persons with MS and healthcare providers about benefits of smoking cessation.

Our AF for smoking in MS of 10% to 15% is lower than for other conditions, including lung cancer. (6) However, this proportion, as well as absolute numbers behind it, is nevertheless substantial. Taking the conservative AF of 13%, a minimum 364,000 of the 2.8 million MS cases worldwide could potentially have been prevented. Considering that fear of long-term disability associated with neurological disease is for many people more prominent than fear of cancer, (19) MS is probably a strong deterrent to smoking initiation. Avoidance and prevention-focused educational campaigns may be more successful than cessation campaigns.

This, and the fact that smoking preventability is more achievable than prevention of other risk factors such as EBV infection or its consequence, infectious mononucleosis, qualifies smoking prevention as a key strategy in preventing MS.

In addition, many more MS cases, even if unpreventable, would be likely to have a milder course, given the contribution of smoking to disease severity and progression.

In conclusion, integrated efforts need to be aimed not only at smoking cessation but crucially also at smoking prevention. The former will make relapses and disability progression in MS in part preventable, while the latter will make a substantial proportion of MS a preventable disease.

## Data Availability Statement

Data related to the current article are available from Ingrid Kockum, Karolinska Institutet. To be able to share data, a data transfer agreement needs to be completed between Karolinska Institutet and the institution requesting data access. This is in accordance with the data protection legislation in Europe (General Data Protection Regulation [GDPR]). Persons interested in obtaining access to the data should contact AM (ali.manouchehrinia@ki.se).

## Ethics Statement

The studies involving human participants were reviewed and approved by Stockholm ethical regional board at Karolinska Institutet. The patients/participants provided their written informed consent to participate in this study.

## Author Contributions

AM analyzed and interpreted data and wrote and revised the manuscript. JHu analyzed and interpreted data and wrote and revised the manuscript. JHi generated and interpreted data and revised the manuscript. TO generated and interpreted data and revised the manuscript. LA generated and interpreted data and revised the manuscript. IK generated and interpreted data and revised the manuscript. CC wrote and revised the manuscript. All authors contributed to the article and approved the submitted version.

## Conflict of Interest

The authors declare that the research was conducted in the absence of any commercial or financial relationships that could be construed as a potential conflict of interest.

## Publisher’s Note

All claims expressed in this article are solely those of the authors and do not necessarily represent those of their affiliated organizations, or those of the publisher, the editors and the reviewers. Any product that may be evaluated in this article, or claim that may be made by its manufacturer, is not guaranteed or endorsed by the publisher.
